# Beneficial Effects of an Alternating High- Fat Dietary Regimen on Systemic Insulin Resistance, Hepatic and Renal Inflammation and Renal Function

**DOI:** 10.1371/journal.pone.0045866

**Published:** 2012-09-25

**Authors:** Gopala K. Yakala, Roel van der Heijden, Grietje Molema, Martin Schipper, Peter Y. Wielinga, Robert Kleemann, Teake Kooistra, Peter Heeringa

**Affiliations:** 1 Department of Pathology and Medical Biology, University of Groningen, University Medical Center Groningen, Groningen, The Netherlands; 2 TNO-Metabolic Health Research, Leiden, The Netherlands; 3 Top Institute Food and Nutrition, Wageningen, The Netherlands; College of Tropical Agriculture and Human Resources, University of Hawaii, United States of America

## Abstract

**Background:**

An Alternating high- cholesterol dietary regimen has proven to be beneficial when compared to daily high- cholesterol feeding. In the current study we explored whether the same strategy is applicable to a high- fat dietary regimen.

**Objective:**

To investigate whether an alternating high- fat dietary regimen can effectively diminish insulin resistance, hepatic and renal inflammation and renal dysfunction as compared to a continuous high- fat diet.

**Design:**

Four groups of male ApoE*3Leiden mice (n = 15) were exposed to different diet regimens for 20 weeks as follows: Group 1: low- fat diet (10 kcal% fat); Group 2: intermediate- fat diet (25 kcal% fat); Group 3: high- fat diet (45 kcal% fat) and Group 4: alternating- fat diet (10 kcal% fat for 4 days and 45 kcal% fat for 3 days in a week).

**Results:**

Compared to high fat diet feeding, the alternating and intermediate- fat diet groups had reduced body weight gain and did not develop insulin resistance or albuminuria. In addition, in the alternating and intermediate- fat diet groups, parameters of tissue inflammation were markedly reduced compared to high fat diet fed mice.

**Conclusion:**

Both alternating and intermediate- fat feeding were beneficial in terms of reducing body weight gain, insulin resistance, hepatic and renal inflammation and renal dysfunction. Thus beneficial effects of alternating feeding regimens on cardiometabolic risk factors are not only applicable for cholesterol containing diets but can be extended to diets high in fat content.

## Introduction

In recent decades the prevalence of obesity and overweight has increased tremendously worldwide. This is a major health issue because obesity and overweight are important risk factors for cardiovascular disease (CVD), insulin resistance, and type 2 diabetes mellitus (T2DM) [1], [2].

Obesity develops when an imbalance exists between calories consumed and calories burned and is linked to increased consumption of energy dense foods high in fats and sugars in combination with reduced physical activity [3], [4]. Strict dietary interventions need to be implemented to reduce obesity and its related complications. An effective dietary intervention to improve health is a caloric restriction diet which aims to reduce caloric intake to a level 15–40% lower than is typical. This approach was found to reduce cardiovascular risk factors and improve insulin sensitivity [5], [6]. More recently, an alternative to the caloric restriction diet, an alternate day fasting regimen, also has shown promising results [7].

Despite the beneficial health effects of caloric restriction, most individuals find it difficult to comply with such strict regimens. Therefore, alternative forms of dietary interventions are desired that are more easy to adapt to but have similar beneficial health effects. Recently, we showed that alternating dietary composition can effectively modulate metabolic and cardiovascular risk factors. In this study, we demonstrated that feeding ApoE*3Leiden(E3L) mice with an alternating high-cholesterol (3 days/week) and a cholesterol-free western type diet (4 days/week) for 16 weeks, strongly diminished hepatic, vascular and renal activation and inflammation, and mimicked most of the beneficial effects of a continuous cholesterol-free dietary regimen [8].

The above dietary cholesterol study provided evidence for the concept of alternating dietary regimen as a strategy to diminish detrimental effects of an unhealthy diet. It is unknown whether the same principle is applicable to dietary factors other than cholesterol. To address this question, the current study was designed to test whether an alternating diet strategy would also be beneficial for fat- containing diets wherein high fat diet is alternated with low fat diet. Previously, it has been shown that feeding E3L male mice on a western type high- fat diet leads to insulin resistance and inflammation in both liver and adipose tissue [9], [10]. In the current study, we investigated whether an alternating low- fat (4 days)/high- fat (3 days) dietary regimen was able to diminish the adverse effects caused by a continuous high- fat diet in E3L mice.

## Materials and Methods

### Animals and Diets

Animal experiments were performed according to the criteria outlined in the “Guide for the Care and Use of Laboratory Animals” published by the National Institutes of Health (NIH) and were approved by the University of Groningen, ethical committee on animal care and experimentation (DEC no: 6141 A). Male ApoE*3-Leiden transgenic (E3L) mice (n = 60) were obtained from TNO-Metabolic Health Research, Gaubius Laboratory, Leiden, The Netherlands. Mice (12–15 weeks old) were group housed (3–5 mice per cage) with a 12 h light-dark cycle and had access to water and diet *ad libitum*. Prior to their respective fat diet treatments, all animals received a lard fat diet containing 10 kcal% from fat (low- fat diet (LFD), (D11032101, Research Diets, NJ, USA).

**Figure 1:**
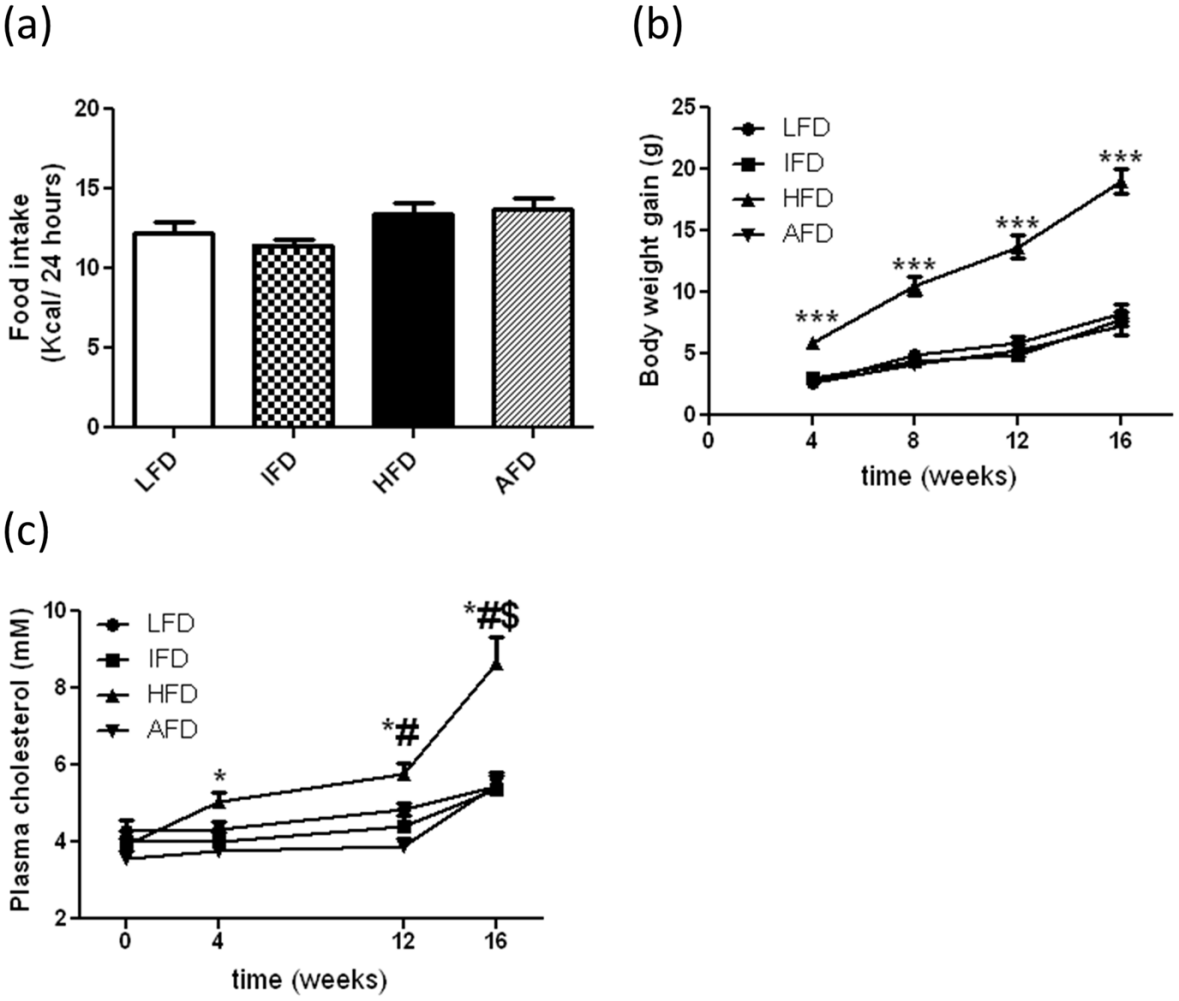
Effect of alternate high-fat dietary regimen on body weight, food intake and plasma cholesterol. (a) Food intake, (b) body weight gain, and (c) plasma cholesterol. Groups are abbreviated as: Mice fed low- fat diet (LFD, n = 15); mice fed intermediate- fat diet (IFD, n = 15); mice fed high- fat diet (HFD, n = 15), and mice fed 4 days LFD and 3 days HFD, alternating- fat diet (AFD, n = 15). ***p<0.001, *p<0.05 compared to LFD, # p<0.05 compared to IFD and $ p<0.05 compared to AFD. Values are represented as means ± SEM.

### Study Design

A schematic representation of the study design including blood and urine collection is depicted in Figure S1. Prior to the start of the experimental treatment period, mice were subjected to an oral glucose tolerance test (OGTT) at t = −2 weeks. After a recovery period of 2 weeks, animals were individually housed in metabolic cages and 24 hour urine was collected. Animals were randomly divided into four groups of 15 animals each and all mice in different groups were treated similarly in terms of handling, and stress to exposures. Technicians performing the analysis were blinded to treatment groups. Group 1 continued on the LFD until the end of the experiment. Group 2 received a lard fat diet containing 25 kcal% from fat (Intermediate- fat (IFD), (D11032102, Research Diets, NJ, USA). Group 3 received a lard fat diet containing 45 kcal% from fat (High- fat diet (HFD), (D11032103, Research Diets, NJ, USA). Group 4 was subjected to an alternating diet regimen (Alternating- fat diet (AFD)) constituting of four days of LFD, followed by three days of HFD in a week. The composition of the different diets used in this study is provided in Table S1. The difference in dietary fat content was compensated with carbohydrates while the other dietary components were similar in all diets.

**Figure 2:**
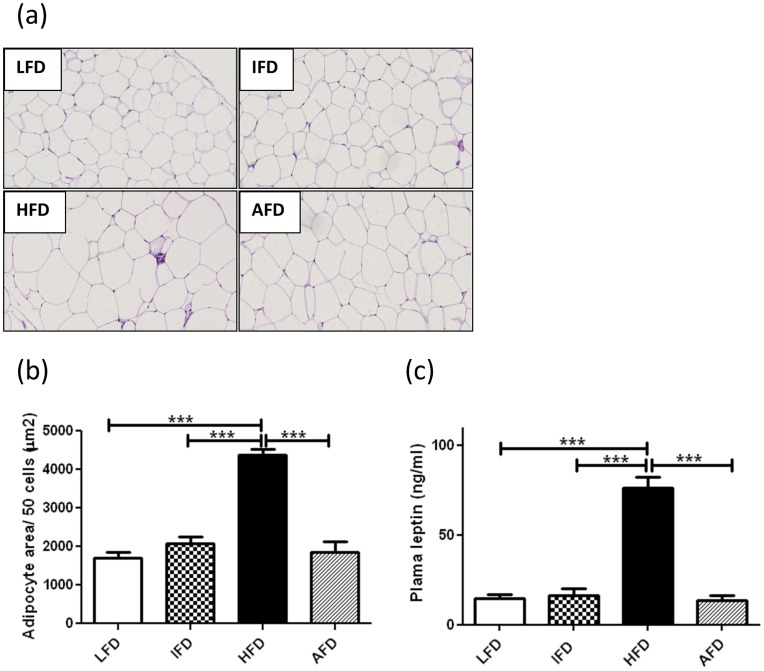
Effect of alternate high-fat dietary regimen on adipocyte size and plasma leptin production. (a) Adipocyte morphology (200×), (b) quantification of the adipocyte size (n = 6 animals/group) and (c) plasma leptin levels (week 16) (c). Groups are abbreviated as: Mice fed low- fat diet (LFD); mice fed intermediate- fat diet (IFD); mice fed high- fat diet (HFD) and mice fed 4 days LFD and 3 days HFD, alternating- fat diet (AFD). ***p<0.001. Values are represented as means ± SEM.

**Figure 3:**
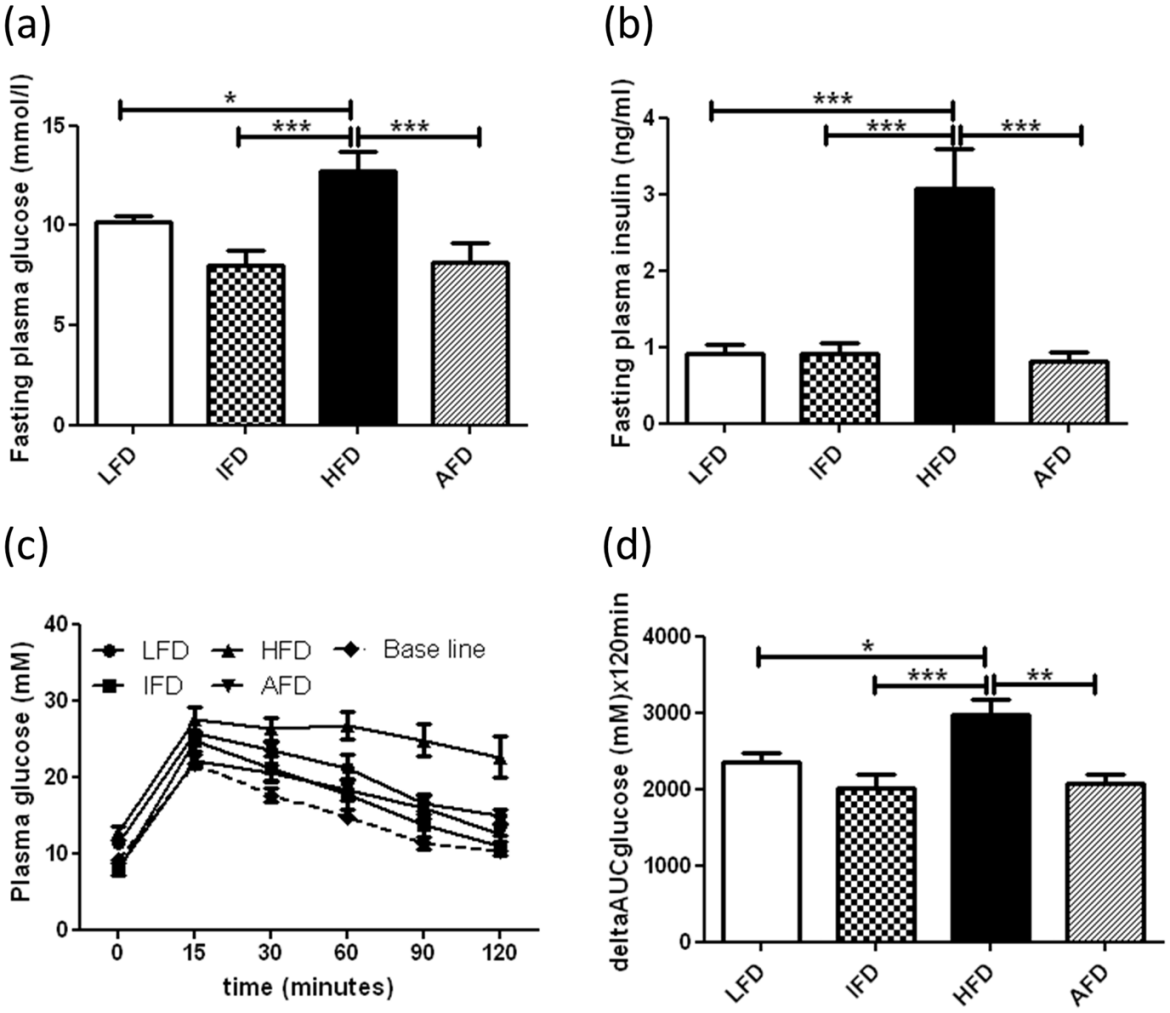
Effect of alternate high-fat dietary regimen on fasting plasma glucose, fasting plasma insulin, and glucose intolerance after 18 weeks. (a) Fasting plasma glucose (n = 8 animals/group), (b) fasting plasma insulin (n = 15 animals/group), (c) glucose tolerance (n = 8 animals/group), and (d) Area under curve of the glucose tolerance test. Groups are abbreviated as: Mice fed low- fat diet (LFD); mice fed intermediate- fat diet (IFD); mice fed high- fat diet (HFD) and mice fed 4 days LFD and 3 days HFD, alternating- fat diet (AFD)). Lines with regular ticks indicate base line levels of the glucose clearance from blood. *p<0.05, **p<0.01, ***p<0.001. Values are represented as means ± SEM.

Body weight (individually, every 2 weeks) and food intake (at cage level, every 8 weeks) were monitored and blood samples were taken by orbital puncture under anesthesia after 4 hours of fasting at t = 0 and weeks 4, 8, 12 and 16. At week 18, animals were subjected to OGTT and monitored for glucose clearance from blood. At weeks 10 and 19, animals were housed individually in metabolic cages to collect 24 hour urine. After twenty weeks of the experimental diet feeding, mice were fasted for 4 hours and sacrificed by Isofluorane/CO_2_ and organs were isolated and weighed. Adipose tissues, aortas, livers (median lobe) and kidneys (left) were fixed and embedded in paraffin; livers (sinister and caudate lobes) and kidneys (right) were snap frozen in liquid N_2_ and stored at −80°C until further use. We considered week 16 as the end point for both body weight and plasma analysis to avoid a possible influence of stress the mice may have experience due to OGTT and 24 hour urine collection performed after 16 weeks.

**Figure 4:**
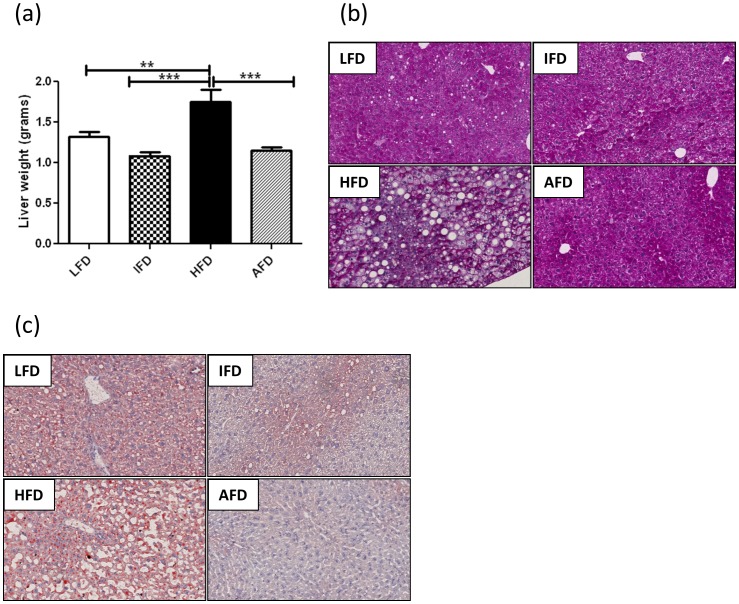
Effect of alternate high-fat dietary regimen on lipid accumulation after 20 weeks. (a) Liver weight, (b) PAS staining of the liver, and (c) Oil Red O (ORO) staining in the liver (200×). Groups are abbreviated as: Mice fed low- fat diet (LFD); mice fed intermediate- fat diet (IFD); mice fed high- fat diet (HFD) and mice fed 4 days LFD and 3 days HFD, alternating- fat diet (AFD). **p<0.01, ***p<0.001. Values are represented as means ± SEM.

### Oral Glucose Tolerance Test

Glucose tolerance tests were performed after a 5h-fasting period. Mice were injected with a bolus of glucose (2 g/kg body weight, 20% glucose in water) orally and blood glucose levels were monitored for 120 minutes using a hand-held glucose analyzer (LifeScan, Milpitas, CA, USA) at t = 0 min, 15 min, 30 min, 60 min, and 120 min after glucose injection.

**Table 1:** Relative mRNA expression levels of proinflammatory genes.

Hepatic gene expression profiling				
	LFD (n = 14)	HFD (n = 14)	IFD (n = 14)	AFD (n = 13)
VCAM-1	1.00±0.48^ a^	2.06±0.85^ b^	1.50±1.20^ ab^	1.61±0.55^ ab^
ICAM-1	1.00±0.44^ a^	1.84±0.62^ b^	1.28±0.86^ ac^	1.50±0.32^ bc^
Endothelin-1	1.00±0.50^ a^	1.55±0.49^ b^	1.29±0.73^ ab^	1.10±0.53^ a^
CD68	1.00±0.52^ a^	1.35±0.39 ^b^	1.03±0.53^ ab^	0.91±0.22^ a^
**Renal gene expression profiling**				
	**LFD (n = 14)**	**HFD (n = 14)**	**IFD (n = 14)**	**AFD (n = 13)**
VCAM-1	1.00±0.24 ^a^	1.60±0.88^ b^	1.34±0.58^ ab^	1.43±0.31^ ab^
ICAM-1	1.00±0.27^ a^	1.46±0.33^ b^	1.45±0.58^ ab^	1.69±0.53^ b^
E-selectin	1.00±0.51^ a^	1.48±0.58^ ab^	1.61±1.06^ ab^	2.56±1.75^ b^
Endothelin-1	1.00±0.26^ a^	1.91±1.01^ b^	0.82±0.43^ a^	1.17±0.28^ a^
CD68	1.00±0.34^ a^	1.46±0.76^ b^	1.34±0.54^ ab^	0.84±0.25^ ac^
MCP-1	1.00±0.57^ a^	2.25±2.12^ b^	1.07±0.57^ a^	1.24±0.56^ a^
PDGF-b	1.00±0.20^ a^	1.43±0.48^ b^	1.11±0.30^ a^	1.31±0.22^ ab^
Desmin	1.00±0.22^ a^	1.45±0.47^ b^	1.10±0.42^ a^	1.22±0.31^ a^

Hepatic mRNA expression of endothelial activation (VCAM-1, ICAM-1 and endothelin-1) and inflammation markers (CD68). Renal mRNA expression of endothelial activation (VCAM-1, ICAM-1, E-selectin and Endothelin-1), inflammation (MCP-1 and CD68), and fibrosis markers (Desmin and PDGF-b). Groups are abbreviated as: Mice fed low- fat diet (LFD); mice fed intermediate- fat diet (IFD); mice fed high- fat diet (HFD) and mice fed 4 days LFD and 3 days HFD, alternating- fat diet (AFD). Superscripts without a common letter differ significantly P<0.05. Values are represented as means ± SD.

### Analysis of Plasma Lipids

Total plasma cholesterol and triglyceride levels were measured after 4 hours of fasting; using commercial kits (catalogue No. 11489437 and 11488872, respectively; Roche Diagnostics, Almere, The Netherlands). The plasma levels of insulin (Alpco, Tilburg, The Netherlands) were determined by ELISA according to the manufacturer’s instructions.

**Figure 5:**
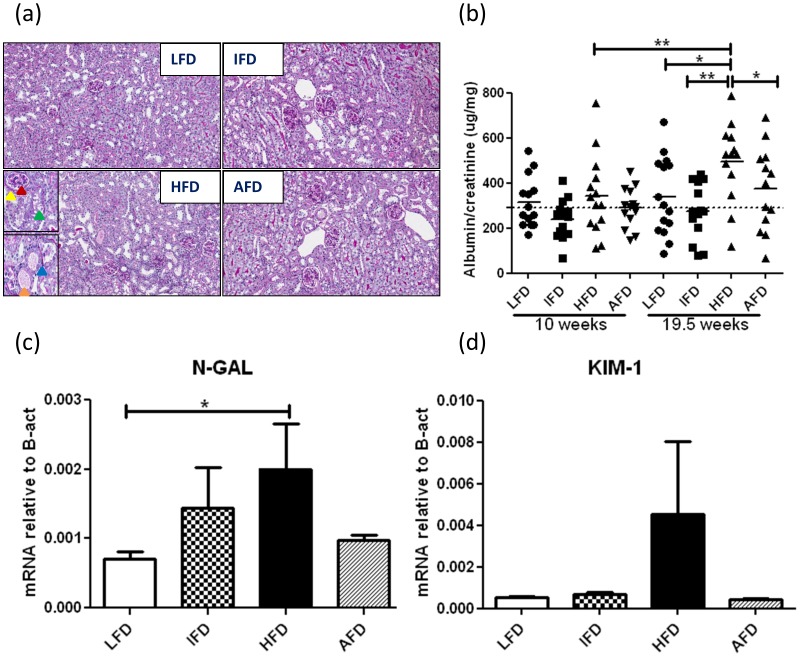
Effect of alternate high-fat dietary regimen on renal morphology and function. (a) Morphology of the kidney (PAS staining). Mesangial expansion (PAS positive area) (red arrow), thickening of the Bowman ’s capsule (yellow arrow), thickening of tubular basement membrane (blue arrow), vacuolarization (lipid accumulation) of the tubules (green arrow), and protein cast formation (orange arrow) (inset- highlighted areas in HFD kidney) (200×) (b) Albumin: creatinine ratio (n = 13−15). Lines with regular ticks indicate base line levels. (c) relative mRNA expression levels of NGAL, (d) relative mRNA expression levels of KIM-1. Groups are abbreviated as: Mice fed low- fat diet (LFD); mice fed intermediate- fat diet (IFD); mice fed high- fat diet (HFD) and mice fed 4 days LFD and 3 days HFD, alternating- fat diet (AFD). *p<0.05, **p<0.01. Values represented as individual animal + mean.

### Renal and Hepatic RNA Extraction and Gene Expression Analysis

Total RNA was extracted from thirty 5- µm thin cryo-sections from both kidney and liver using RNeasy Mini plus Kit (Qiagen, Westburg, Leusden, The Netherlands) according to the manufacturer’s instructions. Integrity of RNA was determined by Agarose gel electrophoresis. RNA quantity (OD-260) and quality (OD-260/OD-280) were determined using an ND-1,000 UV-Vis spectrophotometer (NanoDrop Technologies, Rockland, DE).

**Figure 6:**
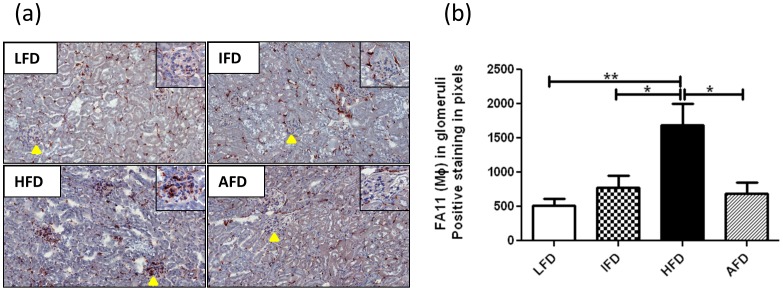
Effect of alternate high-fat dietary regimen on renal inflammation. (a) Immunohistochemistry of macrophage infiltrates. Inset shows a representative glomerulus which is indicated by arrow mark (200×), and (b) quantification of macrophage infiltrates in glomeruli (n = 8 animals/group). Groups are abbreviated as: Mice fed low- fat diet (LFD); mice fed intermediate- fat diet (IFD); mice fed high- fat diet (HFD) and mice fed 4 days LFD and 3 days HFD, alternating- fat diet (AFD). *p<0.05, **p<0.01. Values are represented as means ± SEM.

Total RNA was reverse-transcribed using SuperScript III reverse transcriptase (Invitrogen, Breda, The Netherlands) and random hexamer primers (Promega, Leiden, The Netherlands). To detect the expression of selected target genes Assays-On-Demand™ gene expression primer/probe sets (ABI Systems, Foster City, CA) were used. Endogenous PPIA (assay ID Mm02342430_g1) and B-actin (assay Mm01205647_g1) were used as housekeeping genes along with the following probes: CD68 (assay IDMm00839636_g1), monocyte chemotactic protein-1 (MCP-1; assay IDMm00441242_m1), VCAM-1 (assay IDMm00449197_m1), inter-cellular adhesion molecule 1 (ICAM-1, assay IDMm00516023_m1), Endothelin-1 (assay IDMm00438656_m1), E-selectin (assay IDMm00441278_m1), Desmin (assay Mm01205647_g1), Neutrophil gelatinase-associated lipocalin (NGAL, assay ID Mm01324470_m1), Kidney Injury Molecule 1 (KIM-1, assay IDMm00506686_m1), and PDGF-b (assay Mm00440678_m1). Real-time PCR was performed in duplicate and the obtained threshold cycle (CT) values were averaged. Relative mRNA levels were calculated as 2^−ΔCT^, in which ΔCT is CT_ gene of interest_ – CT_ housekeeping gene_.

### Histology and Immunohistochemistry

For light microscopy, 5 µm epididymal fat tissue and 3 µm renal paraffin sections were stained with Periodic acid-Schiff’s (PAS). In short, paraffin sections were deparaffinized and re-hydrated in distilled water. Sections were placed in 0.5% periodic acid solution for 5 minutes. After rinsing in distilled water, sections were incubated in Schiff’s reagent (Sigma) for 15 minutes followed by rinsing in lukewarm water for 5 minutes. Sections were counterstained with Mayer’s hematoxylin for 1 minute and washed in tap water. Immunohistochemical staining for macrophages, E-selectin and VCAM-1 were performed on acetone-fixed 5 µm cryosections using an anti-rabbit peroxidase-based Envision®+ system (DakoCytomation, Carpinteria, CA, USA). Briefly, sections were incubated for 60 min with 5 µg/ml rat-anti-mouse CD68 (clone FA11, serotech, Puchheim, Germany), 10 µg/ml rat-anti-mouse E-selectin (MES-1, kind gift from Dr.D.Brown, Celltech group (Slough, UK)) and 10 µg/ml rat-anti-mouse VCAM-1 (Clone MK 2.7, American type Culture Collection (Virginia, USA)) followed by a 30 min incubation with 10 µg/ml unlabeled rabbit-anti-rat secondary antibody (Vector Laboratories, Burlingame, CA, USA). After detection of peroxidase activity with 3-amino-9-ethylcarbazole, sections were counterstained with Mayer’s hematoxylin. Frozen sections were used for oil-red O staining to evaluate accumulation of neutral lipids in the liver and kidney. Images were taken with Aperio ScanScope XT (Aperio, Vista, CA, USA) using 200× magnification and the extent of macrophage infiltration in the glomeruli and the diameter of epididymal adipocytes were determined by morphometry in a blinded manner using aperio imagescope IHC analysis algorithm (Aperio, Vista).

### Kidney Function Measured by Albumin/Creatinine Ratio

To assess renal function, the microalbumin and creatinine levels were measured in mouse urine using commercially available kits. Mouse Albumin ELISA Quantitation set (Bethyl laboratories, Montgomery, Texas, USA) and creatinine clearance (Exocell, Philadelphia, PA) according to the manufacturer’s instructions.

### Statistical Analysis

Data were analyzed with Graphpad prism (Graphpad software 5.0, San Diego CA, USA) and SPSS 17.0 for Windows. Changes over time were evaluated with two-way repeated measures ANOVA with factors treatment (between subjects) and time (within subjects) followed by Bonferroni, post-hoc analysis. Differences between groups at one specific time point were analyzed with 1-way ANOVA followed by LSD post-hoc analysis. P<0.05 was considered significant. Results are shown as means ± SEM, unless stated otherwise.

## Results

### Both Alternating and Intermediate-Fat Dietary Regimens Reduce Body Weight Gain and Improve Plasma Lipids

The average food intake expressed as kCal/24 hours did not differ significantly between the groups (Figure 1a). Body weight at baseline (week 0) was 24.0±0.2 g on average of all groups together. Mice that consumed a HFD displayed a gradual increase in body weight over the 16 week diet intervention period (19.0±1.0 g weight gained at week 16). Compared to HFD fed mice, the increase in body weight during the 16 week diet period was significantly lower at all-time points in mice that consumed a LFD, IFD or AFD. (8.3±0. 7 g, 7.8±0.6 g and 7.2±0.7 g for LFD, IFD and AFD, respectively after 16 weeks) (Figure 1b).

Total plasma cholesterol at baseline (week 0) of all groups together was 3.93±0.11 mM. Mice that consumed a HFD showed a gradual increase in plasma cholesterol levels over time. At week 16, plasma cholesterol levels in the HFD group (8.63±0.67 mM) were significantly higher when compared to the LFD (5.42±0.29 mM), the IFD (5.35±0.45 mM) or the AFD (5.46±0.27 mM) groups (Figure 1c).

### Both Alternating and Intermediate-Fat Dietary Regimens Decrease Adipocyte Size and Reduce Leptin Production

Adipocyte size in the epididymal adipose tissue in the HFD group was significantly increased when compared to the LFD, IFD and AFD groups (Figure 2a and 2b). At t = 0 plasma leptin levels were 2.5±0.21 ng/ml on an average of all groups together. At 16 weeks, the HFD group showed a significant increase in plasma leptin levels (76.2±5.9 ng/ml), when compared to the other three groups (LFD 14.7±2.1 ng/ml, IFD 16.4±3.6 ng/ml and AFD 13.3±2.8 ng/ml) (Figure 2c).

### Both Alternating and Intermediate-Fat Dietary Regimens Reduce Fasting Blood Glucose, Plasma Insulin, and Glucose Intolerance

To investigate whether the increase in body weight after high- fat diet consumption was associated with systemic insulin resistance, we determined blood glucose (n = 8/group) and plasma insulin (n = 15/group) levels and performed oral glucose tolerance tests (n = 8/group), two weeks before (week −2 baseline) the start of the dietary interventions and two weeks prior to sacrifice (week 18). No significant differences in fasting blood glucose (average 8.78±0.44 mM) or plasma insulin (average 0.34±0.02 mM) levels were observed at baseline between the different groups. Plasma glucose levels after 18 weeks in the HFD group (12.76±0.90 mM) were significantly higher, when compared to the LFD (10.19±0.29 mM), IFD (7.98±0.79 mM) and AFD (8.13±1.02 mM) groups (Figure 3a). Also, fasting plasma insulin levels in the HFD group (3.07±0.52 ng/ml) were significantly higher when compared to the LFD (0.91±0.12 ng/m)l, IFD (0.91±0.12 ng/ml) and AFD (0.92±0.13 ng/ml) groups (Figure 3b).

At baseline, all mice were able to clear a bolus of glucose within 120 min (Figure 3c, dotted line). However, after 18 weeks, mice on a HFD showed glucose intolerance, whereas mice that had consumed a LFD, IFD or AFD were still able to clear a glucose bolus in 120 min (Figure 3c). The area under curve (AUC) of the HFD group was significantly higher compared to the other three groups (LFD, IFD, and AFD) (Figure 3d).

### Both Alternating and Intermediate-Fat Dietary Regimens Reduce Lipid Accumulation and Expression of Inflammatory Markers in the Liver

At sacrifice, livers were significantly heavier in the HFD group (1.75±0.14 g), when compared to the other three groups (LFD 1.32±0.06 g, IFD 1.08±0.04 g and AFD 1.15±0.04 g) (Figure 4a). Histological analyses demonstrated marked differences in the accumulation of lipid droplets in the liver among the four groups. In the HFD group fatty livers were observed (hepatic steatosis) as evidenced by numerous lipid droplets in the livers of these mice. Livers from mice of the LFD and IFD groups showed fewer lipid droplets whereas in the AFD group only occasionally droplets were observed in the PAS staining (Figure 4b). Consistent with these results, livers from mice of the HFD group showed more extensive oil-red O (ORO) staining compared to the other three groups (Figure 4c).

To investigate the effect of the diets on hepatic inflammation and endothelial activation, we next analyzed mRNA expression levels of various markers (Table 1). Compared to the LFD group, hepatic mRNA expression levels of endothelial activation markers VCAM-1, ICAM-1, and endothelin-1 were significantly upregulated in livers of the HFD group in conjunction with increased mRNA levels of the macrophage marker CD68. Compared to continuous HFD feeding, the AFD group showed decreased expression levels of Endothelin-1 and CD68 whereas in the IFD group no differences were observed in the expression of VCAM-1 and CD68 (Table 1).

### Both Alternating and Intermediate- Fat Dietary Regimens Reduce Renal Inflammation and Improve Renal Function

At sacrifice, no significant differences were found in kidney weights between the groups (data not shown). Light microscopic analysis revealed the development of mild renal abnormalities in mice that had consumed a HFD chronically. This included mesangial area expansion, thickening of Bowman’s capsule and basement membranes, accumulation of lipid droplets in tubuli, and protein cast formation (Figure 5a). Representative examples of Oil Red O staining of renal sections demonstrating abundant positive staining in the kidneys of HFD treated mice compared to LFD, IFD or AFD treated mice (Figure S2). At t = 0 albumin/creatinine levels were 292±110 µg/mg on average for all groups. In mice fed a chronic HFD, urinary albumin/creatinine levels gradually increased reaching statistical significance at the end of the experimental period when compared to the other groups; at 16 weeks: 494±169 µg/mg in HFD, 340±166 µg/mg in LFD, 275±119 µg/mg in IFD and 374±178 µg/mg in AFD (Figure 5b).

Renal mRNA expression levels of various markers for endothelial activation (VCAM-1, ICAM-1, and Endothelin-1), inflammation (MCP-1 and CD68) and fibrosis (Desmin, and PDGF-b) were significantly upregulated in the HFD group compared to the LFD group. Interestingly, the AFD group had markedly decreased expression levels of Endothelin-1, and MCP-1 when compared to the HFD group, and decreased levels of CD68 when compared to the HFD and IFD groups. Furthermore, we observed increased mRNA expression levels of renal injury markers NGAL and KIM-1 in the HFD group compared to other three groups (Figure 5c–d) although statistical significance was only reached for NGAL when compared to the LFD group. Consistent with the mRNA expression levels, immunohistochemical analysis revealed increased expression of VCAM-1, especially in glomeruli, in the HFD group compared to other three groups (Figure S3a). Immunohistochemistry of E-selectin showed restricted glomerular expression in all four groups. However, both HFD and AFD groups showed slightly increased expression compared to LFD and IFD groups (Figure S3b). Increased infiltration of FA11+ macrophages was detected in the kidneys of HFD treated mice which mainly accumulated in glomeruli as quantified by morphometry (Figure 6b). Compared to the HFD group, mice fed an AFD displayed substantially less glomerular macrophage accumulation, which was comparable to the LFD and IFD groups (Figure 6a).

## Discussion

In the current study, we employed a humanized mouse model, the ApoE*3 Leiden (E3L) male mouse, to determine whether an alternating dietary regimen of high and low fat intake reduces metabolic and cardiovascular risk factors as compared to a continuous high fat diet. We showed that by adapting to a diet with pulsed intake of high and low fat diets (4 days low-fat diet and 3 days high- fat diet in a week) instead of a continuous high- fat diet, fasting plasma glucose and insulin levels were reduced and insulin sensitivity is improved. Furthermore, the alternating high- fat dietary regimen replicated most of the beneficial effects instigated by daily consumption of a low- fat or intermediate- fat diet including reduced body weight gain and plasma cholesterol levels, improved adipocyte physiology, reduced hepatic and renal inflammation and improved renal function compared to continuous HFD.

Recently, we reported the beneficial effects of a similar dietary strategy with high cholesterol containing diets (alternating high- cholesterol dietary regimen) on the occurrence of atherosclerosis, hepatic and renal activation and inflammation [8]. This study together with the results presented here and existing literature [7], [8] indicate that the principle of an alternating dietary regimen (ADR) is a strong alternative for some of the inflexible daily calorie restriction strategies available so far.

The exact molecular mechanisms underlying the beneficial effects of the alternating dietary strategy are not yet fully understood. Based on literature and our own findings we speculate that chronic and/or long term exposure to excessive or inappropriate nutrients places a heavy burden on adaptive responses that leads to a gradual loss of metabolic homeostasis and a pattern of continuous, low-grade inflammatory and stress responses, leading ultimately to metabolically driven pathologies [11], [12]. Recently, we showed that feeding E3L male mice a high- fat diet chronically results in increased body weight and plasma lipid content and induces insulin resistance in liver and adipose tissue [9]. In addition, we showed that a high- fat diet leads to hepatic steatosis and changes in adipose tissue physiology [9]. In the current study, we found that an alternating high- fat dietary regimen, similar to low- fat and intermediate- fat diets, significantly diminished these metabolic and functional risk factors as reflected by reduced body weight gain, reduced total plasma cholesterol levels and reduced adipocyte size and leptin production.

In the liver, accumulation of lipids, as observed in mice fed a HFD chronically, is a major cause of oxidative stress and inflammation causing the liver to gain size and weight and inducing hepatic steatosis [3]. Here, we found that mice exposed to an alternating high- fat dietary regimen were protected against the increase in liver weight and the development of hepatic steatosis when compared to daily HFD feeding. Also, AFD fed mice displayed diminished liver inflammation as evidenced by significantly reduced mRNA expression levels of various inflammatory markers although most of these markers were still elevated when compared to the LFD group.

The development of type 2 diabetes is well known to be associated with renal structural and functional changes characterized by renal inflammation and glomerulosclerosis, decline in glomerular filtration rate and the occurrence of proteinuria [13], [14]. Similar to the liver, intrarenal accumulation of lipids has been proposed to play a major role in causing diabetes associated nephropathy [10]. For example, intrarenal lipid accumulation induces glomerular expression of MCP-1 and VCAM-1 [15], [16], which contribute to glomerular macrophage accumulation [8], [17], eventually leading to albuminuria [18]. In the current study, several parameters analyzed were consistent with the development of (early) diabetic nephropathy in HFD fed mice including the development of albuminuria and histological evidence for mesangial area expansion, thickening of Bowman’s capsule and tubular basement membranes [19], [20], tubular lipid accumulation and protein cast formation. These renal structural and functional changes were associated with increased mRNA expression levels of markers for endothelial activation (VCAM-1, ICAM-1, E-selectin and endothelin-1), inflammation (MCP-1 and macrophage marker CD68), fibrosis (desmin and PDGF-b) [13], [19] and renal injury markers (NGAL and KIM-1) [21], in the HFD group compared to the LFD group. Recently, Fu WJ et al., reported an association between glomerular hyperfiltration, elevated tubular injury markers and decline in kidney function in diabetic nephropathy patients [21]. Interestingly, mice subjected to the AFD regimen displayed downregulated expression levels of the endothelial dysfunction marker endothelin-1, the inflammatory markers MCP-1 and CD68 and the fibrosis markers desmin when compared to HFD fed mice.

In our experiments, a group of mice (IFD group) was included that received a 25% (energy) fat diet daily to compare with the overall dietary fat intake of mice exposed to the AFD regimen (4 days 10%, 3 days 45%). For many of the parameters analyzed, the beneficial effects detected in the AFD group were also observed in the IFD group with only minor differences in some parameters (e.g., CD68 mRNA expression in the liver). These data are in line with recent studies by de Wit et al. who demonstrated that increasing the dietary fat (palm oil) content from 10 kcal% to 20 kcal% did not significantly increase body weight gain in C57Bl6 mice. These findings and the data presented here suggest that the metabolic system is able to successfully cope with a certain amount of dietary fat (buffering capacity) without considerably affecting metabolic homeostasis and triggering inflammatory responses [22]. Overall, these results indicate that a threshold exists for metabolic overload, beyond which the body cannot cope with metabolic stress leading to the induction of various abnormalities. Our current study indicates that the threshold under our experimental conditions must be in between the 25 and 45% lard fat diet. Thus, reduction in metabolic stress caused by a high fat diet can be achieved either by giving the body time to recover from the metabolic overload caused by high fat (alternating- fat diet strategy) or by lowering the daily fat intake (Intermediate- fat diet strategy).

In conclusion, this study indicates that the beneficial effects of alternating feeding regimens on metabolic and functional risk factors for CVD are not only applicable for cholesterol containing diets, as we have shown previously, but can be extended to high- fat containing diets. These results provide further support for the concept of alternating dietary regimens as a means to protect against the adverse effects of an unhealthy diet. Additional studies are needed to examine whether the principles of alternating and intermediate dietary regimens can also be implemented to improve existing metabolic diseases. For example, it would be interesting to investigate whether an alternating or intermediate diet can attenuate or reverse the negative effects caused by a long-term HFD consumption in terms of body weight, glucose tolerance and hepatic steatosis. Finally, the ultimate goal is to examine whether the concept of alternating feeding regimens can be translated to humans.

## Supporting Information

Figure S1 **Schematic representation of the feeding regimens**. Illustration of blood and urine collection. The red droplets indicate blood sampling time points, green droplets indicate urine collection time points and blue drop lets indicate blood sampling at the time of oral glucose tolerance test. Groups are abbreviated as: Mice fed low- fat diet (LFD); mice fed intermediate- fat diet (IFD); mice fed high- fat diet (HFD), and mice fed 4 days LFD and 3 days HFD, alternating- fat diet (AFD).
(TIF)

Figure S2 **Effect of alternating high- fat dietary regimen on renal neutral lipid accumulation.** Oil Red O (ORO) staining in the kidney (200×). Groups are abbreviated as: Mice fed low- fat diet (LFD); mice fed intermediate- fat diet (IFD); mice fed high- fat diet (HFD) and mice fed 4 days LFD and 3 days HFD, alternate- fat diet (AFD).
(TIF)

Figure S3 **Effect of alternate high- fat dietary regimen on expression of endothelial adhesion molecules in kidney.** (a) Expression and localization of VCAM-1 and (b) E-selectin (200×). Inset shows a 200× magnification of a representative glomerulus which is indicated by the arrow. Groups are abbreviated as: Mice fed low- fat diet (LFD); mice fed intermediate- fat diet (IFD); mice fed high- fat diet (HFD) and mice fed 4 days LFD and 3 days HFD, alternate- fat diet (AFD).
(TIF)

Table S1 **Composition of the different diets used in this study.** Rodent diets with 10, 25, or 45 kcal% Fat (from Mostly Lard) and with 213 mg Cholesterol/kg Diet.
(DOCX)
