# Supplementary figures and images for: Beneficial Effects of an Alternating High- Fat Dietary Regimen on Systemic Insulin Resistance, Hepatic and Renal Inflammation and Renal Function
Source: PLoS One. 2012 Sep 25;7(9):e45866. doi: 10.1371/journal.pone.0045866 (PMC3458102; doi:10.1371/journal.pone.0045866)

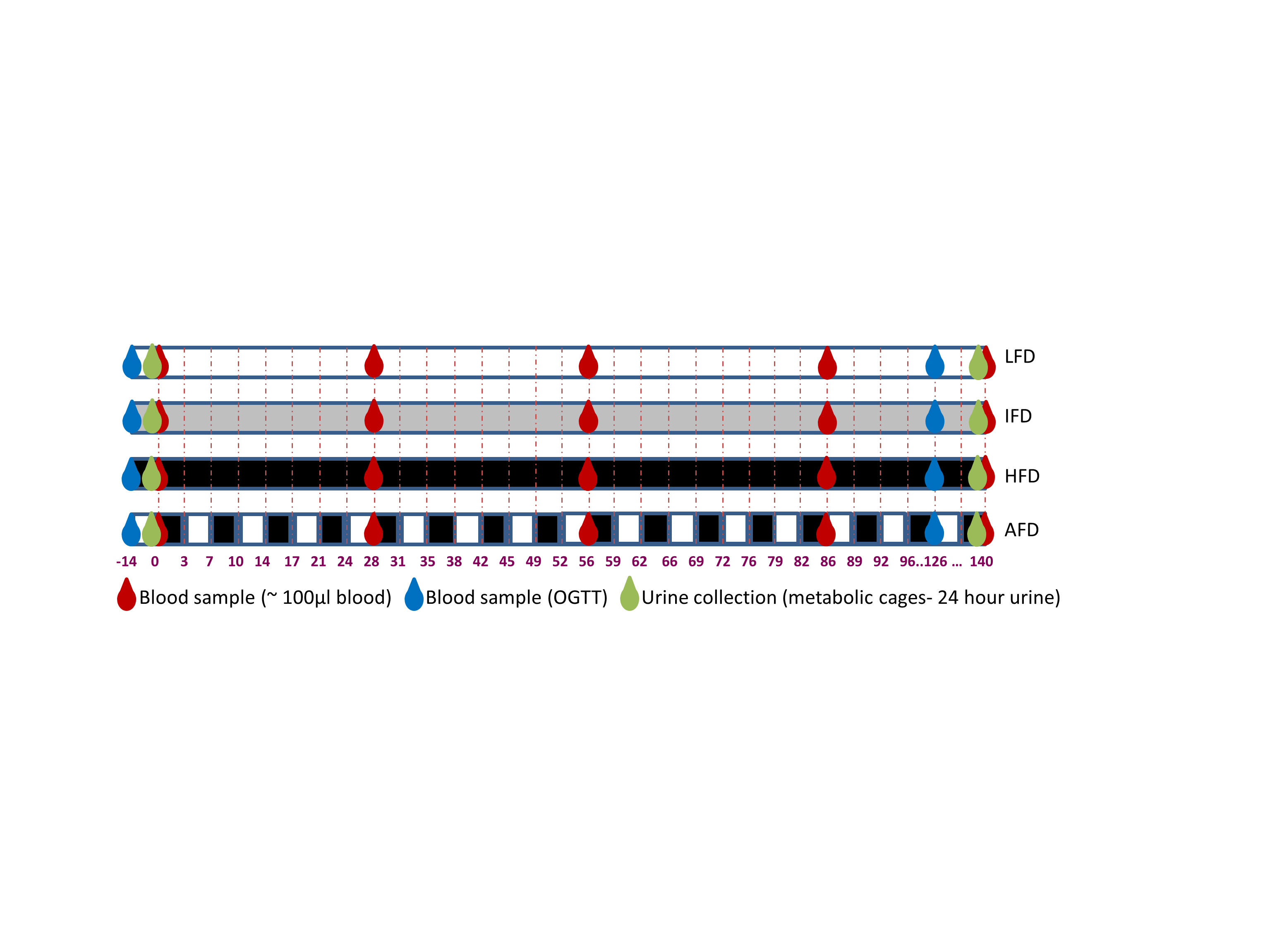

Supplement: Figure S1 — Schematic representation of the feeding regimens. Illustration of blood and urine collection. The red droplets indicate blood sampling time points, green droplets indicate urine collection time points and blue drop lets indicate blood sampling at the time of oral glucose tolerance test. Groups are abbreviated as: Mice fed low- fat diet (LFD); mice fed intermediate- fat diet (IFD); mice fed high- fat diet (HFD), and mice fed 4 days LFD and 3 days HFD, alternating- fat diet (AFD). (TIF) [file pone.0045866.s001.tif]

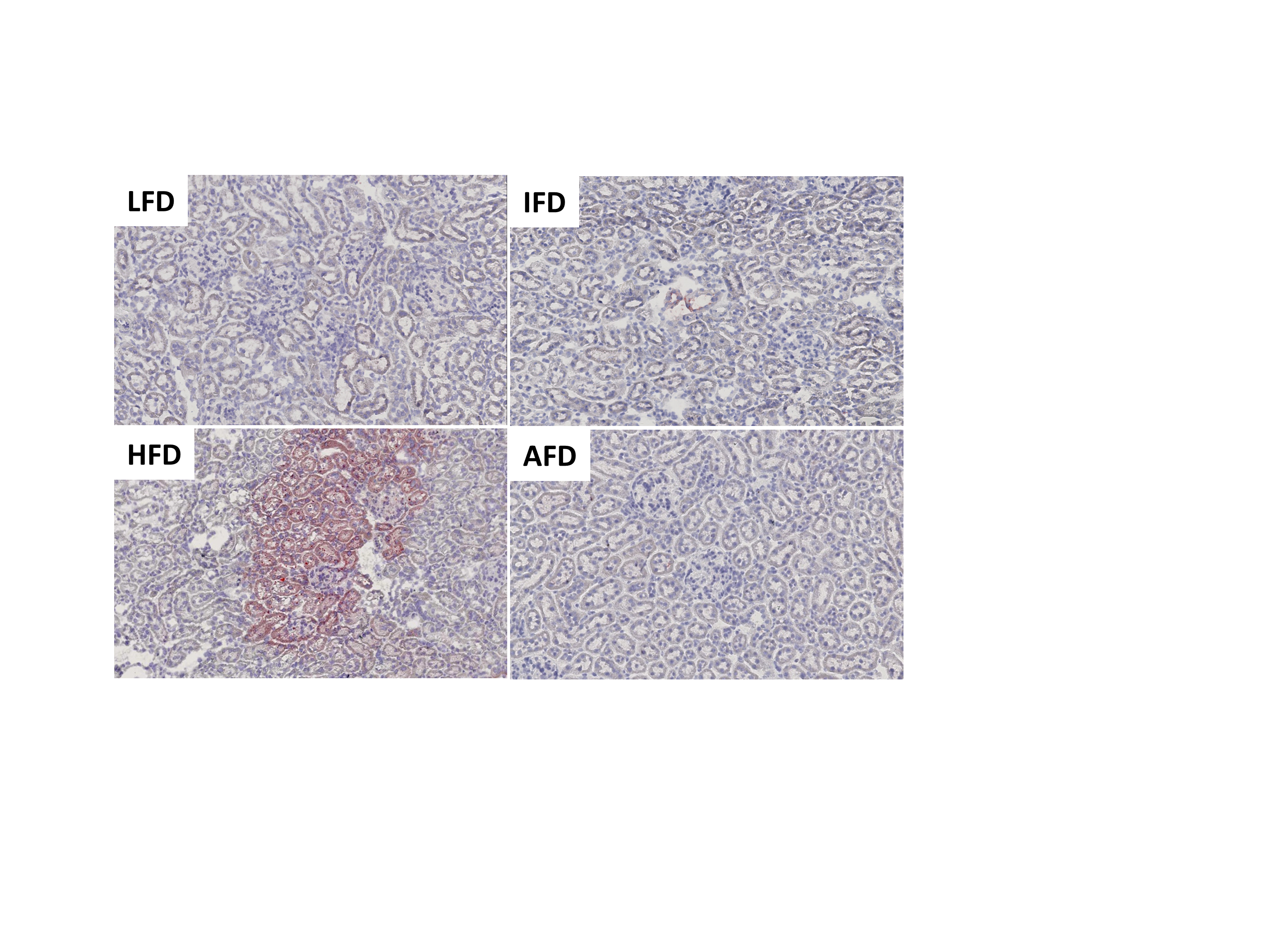

Supplement: Figure S2 — Effect of alternating high- fat dietary regimen on renal neutral lipid accumulation. Oil Red O (ORO) staining in the kidney (200×). Groups are abbreviated as: Mice fed low- fat diet (LFD); mice fed intermediate- fat diet (IFD); mice fed high- fat diet (HFD) and mice fed 4 days LFD and 3 days HFD, alternate- fat diet (AFD). (TIF) [file pone.0045866.s002.tif]

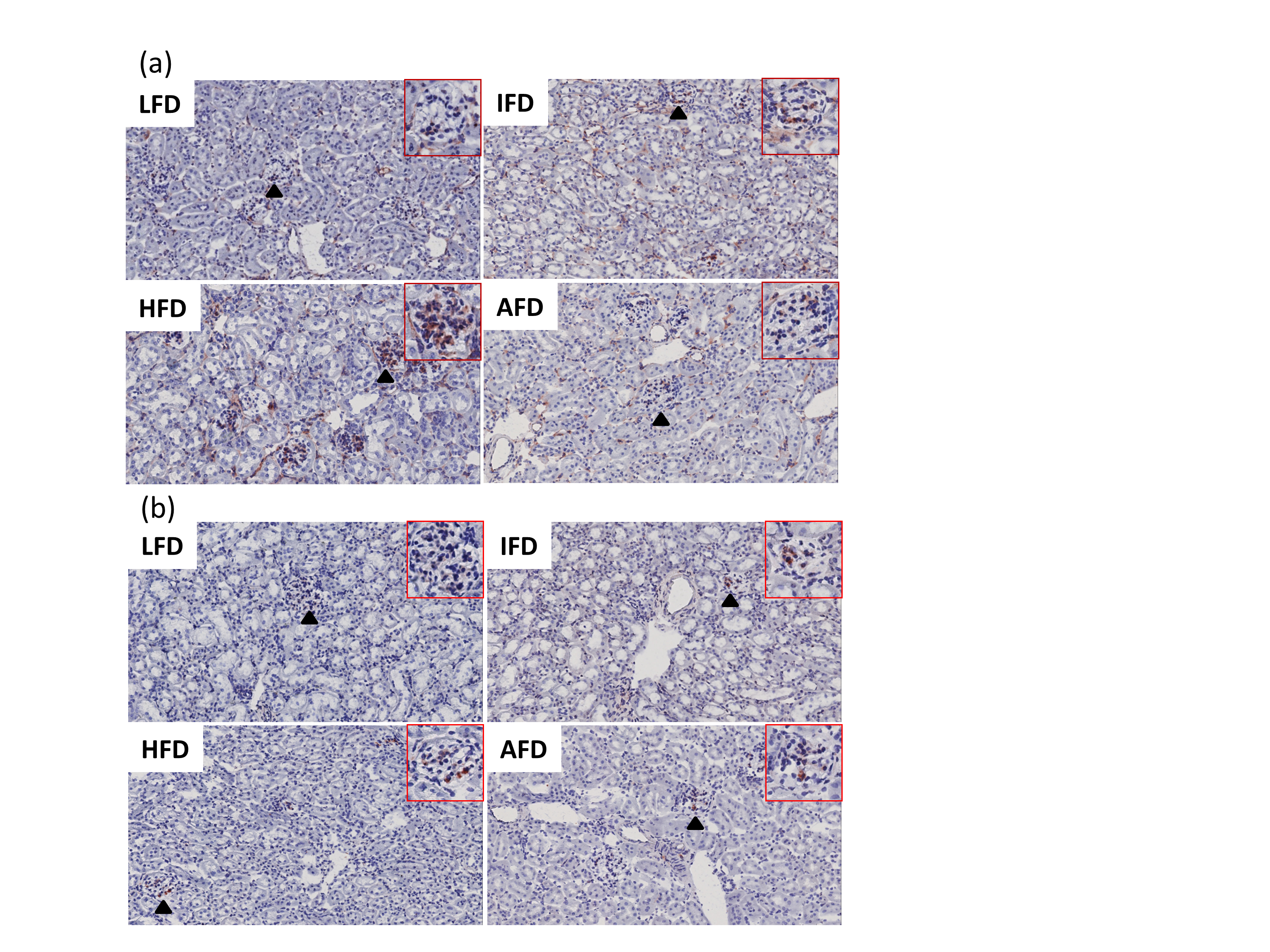

Supplement: Figure S3 — Effect of alternate high- fat dietary regimen on expression of endothelial adhesion molecules in kidney. (a) Expression and localization of VCAM-1 and (b) E-selectin (200×). Inset shows a 200× magnification of a representative glomerulus which is indicated by the arrow. Groups are abbreviated as: Mice fed low- fat diet (LFD); mice fed intermediate- fat diet (IFD); mice fed high- fat diet (HFD) and mice fed 4 days LFD and 3 days HFD, alternate- fat diet (AFD). (TIF) [file pone.0045866.s003.tif]
