# Supplementary material for: Beneficial Effects of an Alternating High- Fat Dietary Regimen on Systemic Insulin Resistance, Hepatic and Renal Inflammation and Renal Function
Source: PLoS One. 2012 Sep 25;7(9):e45866. doi: 10.1371/journal.pone.0045866 (PMC3458102; doi:10.1371/journal.pone.0045866)
Supplement: Table S1 — Composition of the different diets used in this study. Rodent diets with 10, 25, or 45 kcal% Fat (from Mostly Lard) and with 213 mg Cholesterol/kg Diet. (DOCX) [file pone.0045866.s004.docx]

| Product # | D11032101 | | D11032102 | | D11032103 | |
| --- | --- | --- | --- | --- | --- | --- |
| % | gm | kcal | gm | kcal | gm | kcal |
| Protein | 19 | 20 | 21 | 20 | 24 | 20 |
| Carbohydrate | 67 | 70 | 57 | 55 | 41 | 35 |
| Fat | 4 | 10 | 12 | 25 | 24 | 45 |
| Total |  | 100 |  | 100 |  | 100 |
| kcal/gm | 3.8 |  | 4.2 |  | 4.7 |  |
|  |  |  |  |  |  |  |
| Ingredient | gm | kcal | gm | kcal | gm | kcal |
| Casein, 80 Mesh | 200 | 800 | 200 | 800 | 200 | 800 |
| L-Cystine | 3 | 12 | 3 | 12 | 3 | 12 |
|  |  |  |  |  |  |  |
| Corn Starch | 452.2 | 1809 | 272 | 1088 | 72.8 | 291 |
| Maltodextrin 10 | 75 | 300 | 100 | 400 | 100 | 400 |
| Sucrose | 172.8 | 691 | 172.8 | 691 | 172.8 | 691 |
|  |  |  |  |  |  |  |
| Cellulose, BW200 | 50 | 0 | 50 | 0 | 50 | 0 |
|  |  |  |  |  |  |  |
| Soybean Oil | 10 | 90 | 10 | 90 | 10 | 90 |
| Lard | 35 | 315 | 104 | 936 | 192.5 | 1733 |
|  |  |  |  |  |  |  |
| Mineral Mix S10026 | 10 | 0 | 10 | 0 | 10 | 0 |
| DiCalcium Phosphate | 13 | 0 | 13 | 0 | 13 | 0 |
| Calcium Carbonate | 5.5 | 0 | 5.5 | 0 | 5.5 | 0 |
| Potassium Citrate, 1 H2O | 16.5 | 0 | 16.5 | 0 | 16.5 | 0 |
|  |  |  |  |  |  |  |
| Vitamin Mix V10001 | 10 | 40 | 10 | 40 | 10 | 40 |
| Choline Bitartrate | 2 | 0 | 2 | 0 | 2 | 0 |
|  |  |  |  |  |  |  |
| Cholesterol | 0.1815 | 0 | 0.111 | 0 | 0 | 0 |
|  |  |  |  |  |  |  |
| FD&C Yellow Dye #5 | 0.05 | 0 | 0 | 0 | 0 | 0 |
| FD&C Red Dye #40 | 0 | 0 | 0 | 0 | 0.05 | 0 |
| FD&C Blue Dye #1 | 0 | 0 | 0.05 | 0 | 0 | 0 |
|  |  |  |  |  |  |  |
| Total | 1055.23 | 4057 | 968.961 | 4057 | 858.1 | 4057 |

Table S1.
